# Correction: Kitakaze et al. All-*Trans* Retinoic Acid-Responsive LGR6 Is Transiently Expressed during Myogenic Differentiation and Is Required for Myoblast Differentiation and Fusion. *Int. J. Mol. Sci.* 2023, *24*, 9035

**DOI:** 10.3390/ijms26093942

**Published:** 2025-04-22

**Authors:** Tomoya Kitakaze, Rina Tatsumi, Mayu Yamaguchi, Mai Kubota, Aino Nakatsuji, Naoki Harada, Ryoichi Yamaji

**Affiliations:** 1 Division of Applied Life Sciences, Graduate School of Life and Environmental Sciences, Osaka Prefecture University, Sakai 5998531, Osaka, Japan; kitakaze@omu.ac.jp (T.K.); naoki.harada@omu.ac.jp (N.H.); 2 Department of Applied Biological Chemistry, Graduate School of Agriculture, Osaka Metropolitan University, Sakai 5998531, Osaka, Japan; 3 Center for Research and Development of Bioresources, Osaka Metropolitan University, Sakai 5998531, Osaka, Japan

The journal’s Editorial Office and Editorial Board are jointly issuing a resolution and removal of the *Journal Notice* linked to this article [1], as well as an update to the original publication. Following concerns raised about the integrity of one of the peer reviews, the Editorial Office has conducted a post-publication peer review of this article. This process included the recruitment of a new independent reviewer and was supervised by the original Academic Editor to ensure full compliance with MDPI’s Editorial Process (https://www.mdpi.com/editorial_process).

As a result of this process, the Academic Editor and the authors have agreed to update the following aspect of this publication:

- Reviewer report 1 has been removed from the peer review record, and the new report has been uploaded instead (https://www.mdpi.com/1422-0067/24/10/9035/review_report).

Based on the new review report, the following corrections have been made to the original publication:

“Znfr3” has been changed to “Znrf3” in the abstract, “skeletal muscle” has been added to the keywords, and “ZNFR3” has been corrected to “ZNRF3” in Sections 2.4, 2.1, and 2.3 of the Results Section, Section 3 (Discussion), and Sections 4.1 and 4.7 of the Materials and Methods Section.

- New co-author Ms. Mai Kubota was added to perform the additional experiments requested by reviewer 3.
- Data were added to Figure 1G,H in the original publication and are now presented as Figure 1C,D, respectively, in this publication. Accordingly, Figure 1C–F in the original publication are presented as Figure 1E–H, respectively, in this publication.
- Data were added to this publication and are presented in Figure 3E,F. Accordingly, Figure 3E–G in the original publication are now presented as Figure 3G–I, respectively, in this manuscript.
- Data were added to this publication and are presented in Supplementary Figure S3. Furthermore, the text of Section 2.1 in the Results Section was revised. Accordingly, Supplementary Figures S1–S3 in the original publication are now presented as Supplementary Figures S2, S1, and S4, respectively, in this publication.

The following are the corrected figures:

**Figure 1:**
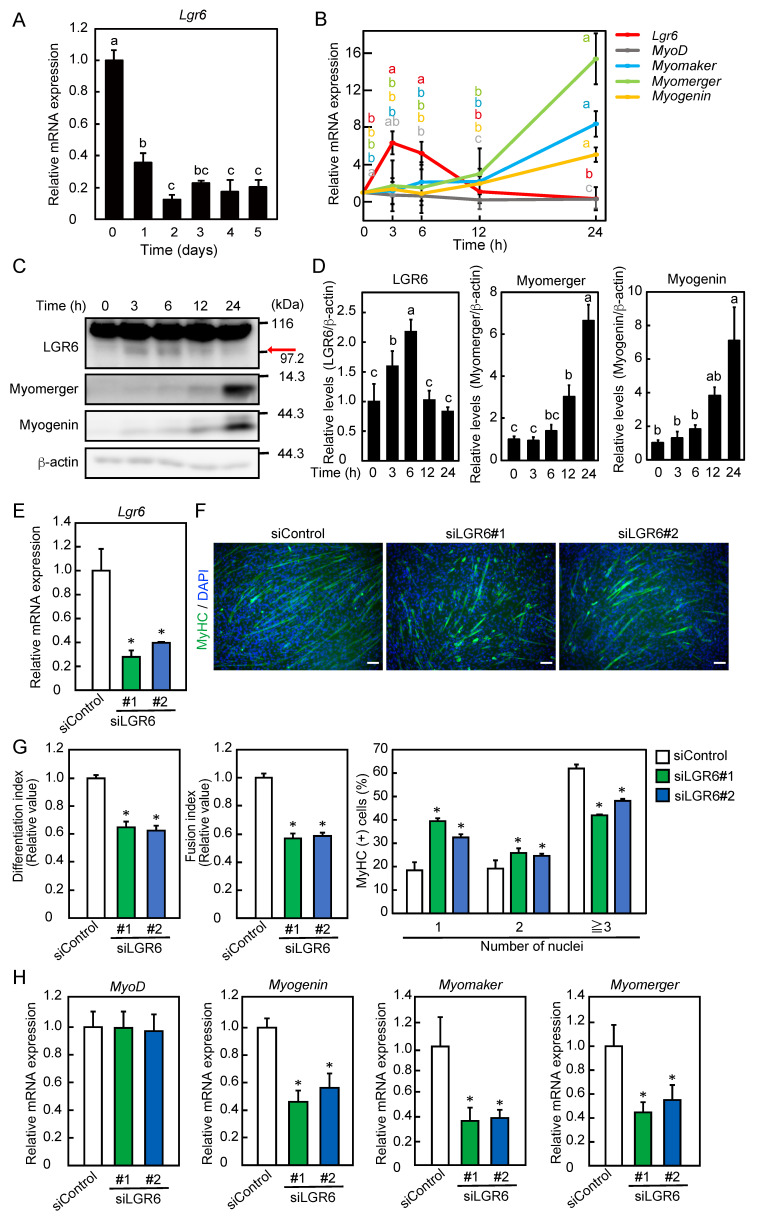
Expression pattern and role of LGR6 during myoblast differentiation. (**A**) C2C12 myoblasts were differentiated into myotubes for 5 days. The expression level of *Lgr6* mRNA was determined by qPCR. (**B**) Myoblasts were differentiated into myotubes for 24 h. The levels of mRNAs encoding LGR6 and myogenic regulatory factors were determined by qPCR. (**C**) Myoblasts were differentiated into myotubes. LGR6, myomerger, and myogenin expression was analyzed by Western blotting. The arrow indicates the lower band of LGR6. (D) LGR6, myomerger, and myogenin levels were normalized to β-actin levels. (**E**) Myoblasts were transfected with control siRNA (siControl) or *Lgr6* siRNA (siLGR6 #1 and siLGR6 #2), and cells were harvested 0 h after the induction of differentiation. The *Lgr6* mRNA levels were determined by qPCR. (**F**) Myoblasts were differentiated for 3 days after siRNA transfection. Fixed cells were fluorescently labeled using an anti-MyHC antibody and fluorescence-labeled secondary antibody (green). The nuclei were stained with DAPI (blue). Bars, 100 μm. (**G**) The differentiation and fusion indices were calculated. The percentage of MyHC-positive cells with one, two, or three more nuclei was determined. (**H**) Myoblasts were differentiated for 24 h after siRNA transfection, and cells were harvested. The levels of mRNAs for myogenic regulatory factors were determined by qPCR. (**A**,**B**,**D**) The results are presented as the mean ± SD (*n* = 3). Data were determined using one-way ANOVA and Tukey’s post hoc test. (**A**,**D**) Columns with different letters are significantly different at *p* < 0.05, whereas columns sharing the same letters are not significantly different. (**B**) Different letters with the same color on the lines indicate statistically significant differences (*p* < 0.05). (**E**,**G**,**H**) The results are presented as the mean ± SD (*n* = 3). Data were determined using one-way ANOVA and Dunnet’s post hoc test. * *p* < 0.05 vs. siControl.

**Figure 3:**
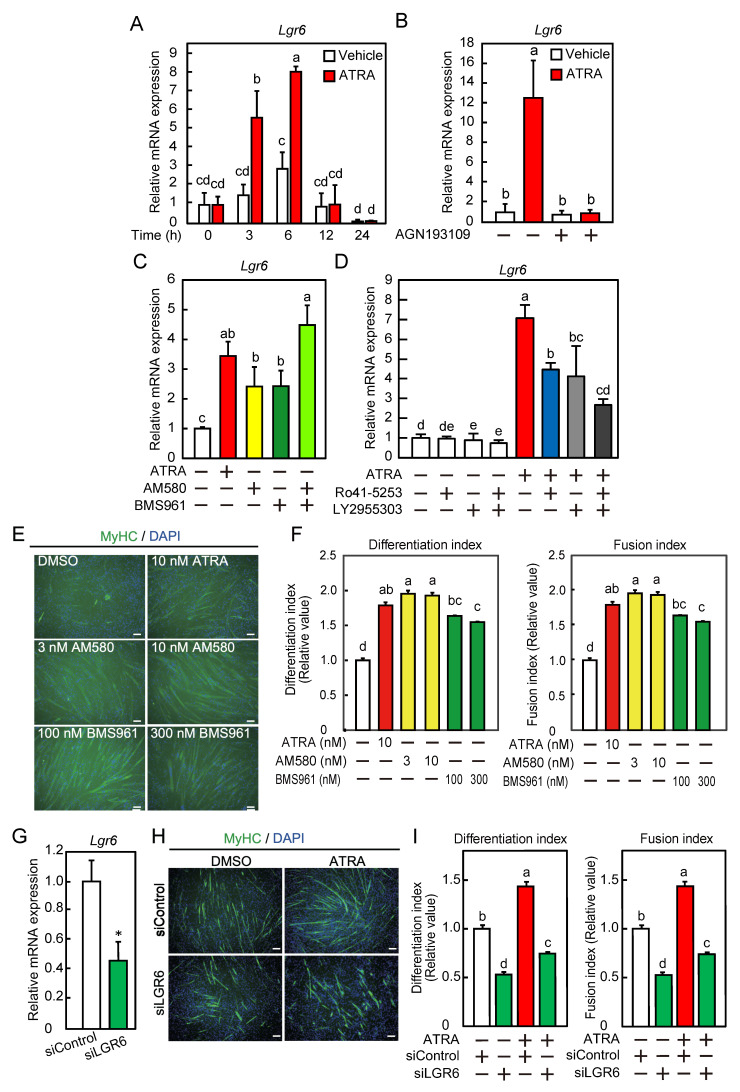
LGR6 expression during myoblast differentiation as an ATRA-responsive gene. (**A**) C2C12 myoblasts were differentiated in the presence or absence of ATRA. The *Lgr6* mRNA levels were determined by qPCR. (**B**) Myoblasts were cultured with ATRA in the presence of AGN193109. The *Lgr6* mRNA levels were determined by qPCR. (**C**) C2C12 myoblasts were cultured with ATRA, AM580, BMS961, AM580, and BMS961. The *Lgr6* mRNA levels were determined by qPCR. (**D**) Myoblasts were cultured with ATRA in the presence or absence of Ro41-5253 and/or LY2955303. The *Lgr6* mRNA levels were determined by qPCR. (**E**) Myoblasts were differentiated in the presence or absence of an RAR agonist. Fixed cells were immunofluorescently labeled using an anti-MyHC antibody (green), and the nuclei were stained with DAPI (blue). Bars, 100 μm. (**F**) The differentiation and fusion indices were calculated. (**G**) Myoblasts were transfected with control siRNA (siControl) or *Lgr6* siRNA (siLGR6#1), and cells were harvested immediately after the induction of differentiation. The *Lgr6* mRNA levels were determined by qPCR. (**H**) After siRNA transfection, myoblasts were differentiated in the presence or absence of ATRA for 3 days. Fixed cells were immunofluorescently labeled using an anti-MyHC antibody (green), and the nuclei were stained with DAPI (blue). Bars, 100 μm. (**I**) The differentiation and fusion indices were calculated. (**A**–**D**,**F**,**I**) The results are presented as the mean ± SD (*n* = 3). Data were determined using two-way ANOVA and Tukey’s post hoc test. Columns with different letters are significantly different at *p* < 0.05, whereas columns sharing the same letters are not significantly different. (**G**) The results are presented as the mean ± SD (*n* = 3). Data were determined using Student’s *t*-test. * *p* < 0.05 vs. siControl.

With this update, the Academic Editor is satisfied that the Editorial Process relating to this article has been completed as per MDPI’s Editorial Process policy. The Editorial Office would like to thank the authors for their collaboration during this process.
