# Supplementary material for: All-Trans Retinoic Acid-Responsive LGR6 Is Transiently Expressed during Myogenic Differentiation and Is Required for Myoblast Differentiation and Fusion
Source: Int J Mol Sci. 2023 May 20;24(10):9035. doi: 10.3390/ijms24109035 (PMC10219391; doi:10.3390/ijms24109035)
Supplement: Supplementary file 1 [file ijms-24-09035-s001.zip › Spplementalry Figure S1.pdf]

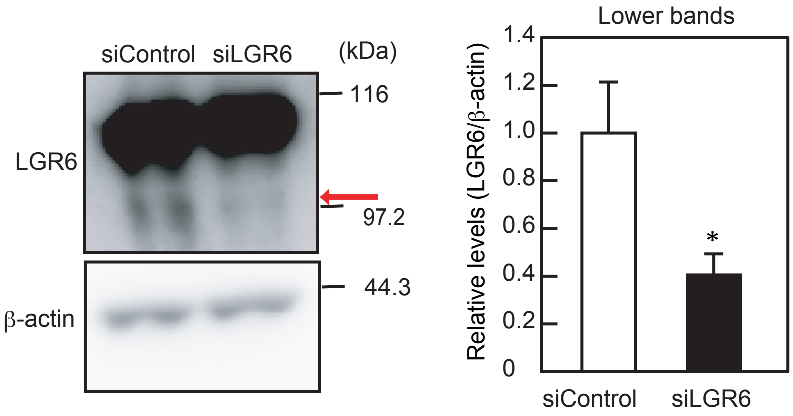

Supplementary Figure S1.
Western blot analysis of LGR6. C2C12 myoblasts were transfected with control siRNA (siControl) or *Lgr6* siRNA (siLGR6 #1) for 24 h, followed by induction of differentiation. Cells were harvested 3 h after the inducion of differentiation. LGR6 expression was analyzed by western blotting, and LGR6 levels were normalized to β-actin levels. Arrow indicates lower band. The results are presented as the mean ± SD ($n = 3$). Data were determined by Student's *t*-test. *$p < 0.05$. vs. siControl.
