# Supplementary material for: All-Trans Retinoic Acid-Responsive LGR6 Is Transiently Expressed during Myogenic Differentiation and Is Required for Myoblast Differentiation and Fusion
Source: Int J Mol Sci. 2023 May 20;24(10):9035. doi: 10.3390/ijms24109035 (PMC10219391; doi:10.3390/ijms24109035)
Supplement: Supplementary file 1 [file ijms-24-09035-s001.zip › Spplementalry Figure S2.pdf]

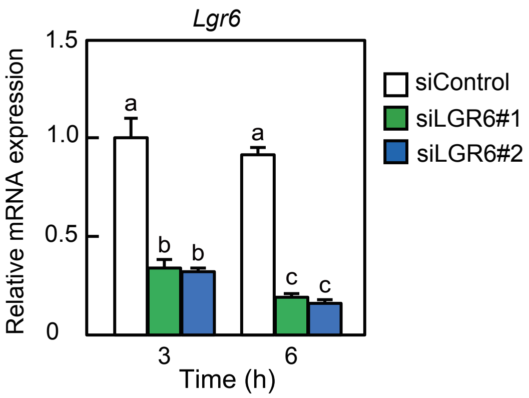

Supplementary Figure S2.
Effects of *Lgr6* siRNA knockdown on *Lgr6* mRNA expression during myogenic differentiation. C2C12 myoblasts were transfected with control siRNA (siControl) or *Lgr6* siRNA (siLGR6 #1 or siLGR6#2) for 24 h, followed by induction of differentiation. Cells were harvested 3 and 6 h after the induction of differentiation. The *Lgr6* mRNA levels were determined by qPCR. The results are presented as the mean ± SD (n = 3). Data were determined by one-way ANOVA and Tukey's post hoc test. Different letters on columns indicate statistically significant differences ($p < 0.05$).
