# Supplementary material for: All-Trans Retinoic Acid-Responsive LGR6 Is Transiently Expressed during Myogenic Differentiation and Is Required for Myoblast Differentiation and Fusion
Source: Int J Mol Sci. 2023 May 20;24(10):9035. doi: 10.3390/ijms24109035 (PMC10219391; doi:10.3390/ijms24109035)
Supplement: Supplementary file 1 [file ijms-24-09035-s001.zip › Supplementary Figure S3.pdf]

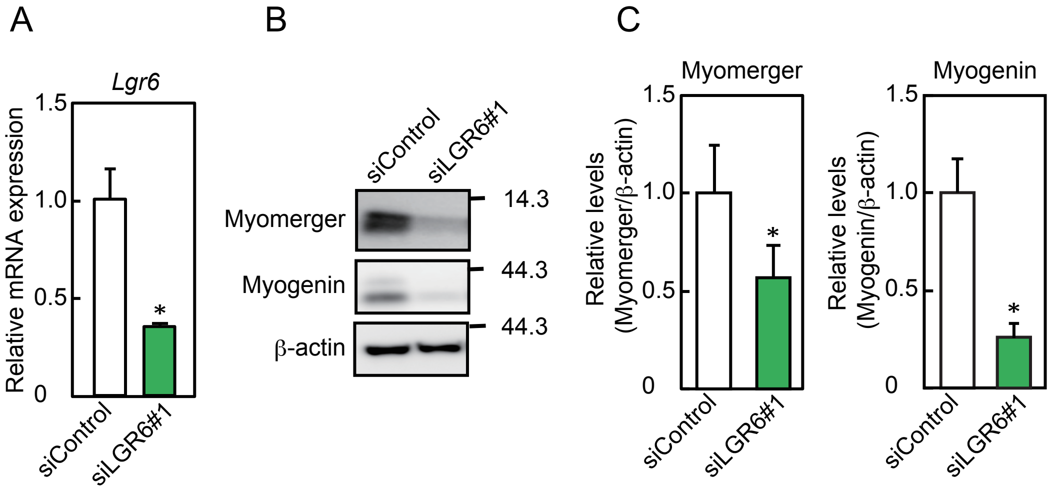

Suppmentary Figure S3.

Western blot analysis of myomerger and myogenin. C2C12 myoblasts were transfected with control siRNA (siControl) or *Lgr6* siRNA (siLGR6 #1) for 24 h, followed by induction of differentiation. (A) Cells were harvested 0 h after the inducion of differentiation. The *Lgr6* mRNA levels were determined by qPCR. The results are presented as the mean ± SD ($n = 3$). The results are presented as the mean ± SD ($n = 3$). Data were determined by Student's *t*-test. *$p < 0.05$. vs. siControl. (B) Cells were harvested 24 h after the induction of differentiation. Myomerger and myogenin expression was analyzed by western blotting. (C) Myomerger and myogenin levels were normalized to β-actin levels. The results are presented as the mean ± SD ($n = 3$). Data were determined by Student's *t*-test. *$p < 0.05$. vs. siControl.
