# Supplementary material for: All-Trans Retinoic Acid-Responsive LGR6 Is Transiently Expressed during Myogenic Differentiation and Is Required for Myoblast Differentiation and Fusion
Source: Int J Mol Sci. 2023 May 20;24(10):9035. doi: 10.3390/ijms24109035 (PMC10219391; doi:10.3390/ijms24109035)
Supplement: Supplementary file 1 [file ijms-24-09035-s001.zip › Supplementary Figure S4.pdf]

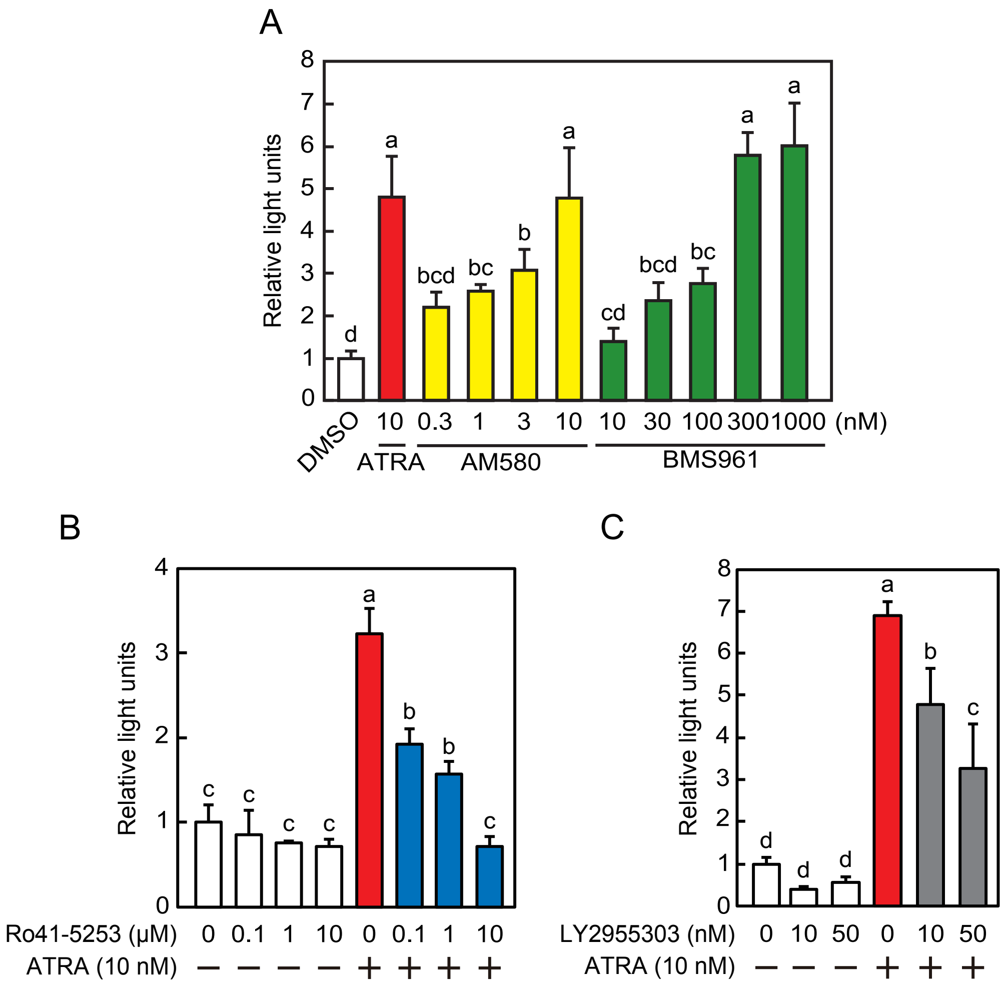

Supplementary Figure S4.

Effects of RAR agonists or RAR antagonists on RAR transcriptional activity. (A) To determine the effects of an RARα agonist (AM580) or an RARγ agonist (BMS961) on RAR transcriptional activity, C2C12 myoblasts were cultured in stripped growth medium and transfected with pGL3-SV40-5×RARE-Luc (reporter vector harboring the retinoic acid response element) and pGL4.75-CMV-hRluc (internal control vector) for 24 h, followed by incubation with vehicle (DMSO), ATRA, AM580, or BMS961 for 24 h. (B) To determine the effects of an RARα antagonist (Ro41-5253) on ATRA-activated RAR transcriptional activity, C2C12 myoblasts were cultured in stripped growth medium and transfected with pGL3-SV40-5×RARE-Luc and pGL4.75-CMV-hRluc for 24 h, followed by incubation with Ro41-5253 in the presence or absence of ATRA for 24 h. (C) To determine the effects of an RARγ antagonist (LY2955303) on ATRA-activated RAR transcriptional activity, C2C12 myoblasts were cultured in stripped growth medium and transfected with pGL3-SV40-5×RARE-Luc and pGL4.75-CMV-hRluc for 24 h, followed by incubation with LY2955303 in the presence or absence of ATRA for 24 h. (A–C) The cells were lysed, and firefly and *Renilla* luciferase activities were determined. Transfection efficiency was normalized using pGL4.75-CMV-hRluc. Data are expressed as relative light units (firefly luciferase activity divided by *Renilla* luciferase activity). The results are presented as the mean ± SD ($n = 3$). Data were determined by two-way ANOVA and Tukey's post hoc test. Different letters on columns indicate statistically significant differences ($p < 0.05$).
